# Virtual reality as an assessment tool and a treatment method in the field of addiction

**DOI:** 10.1192/j.eurpsy.2026.10895

**Published:** 2026-07-31

**Authors:** S. Boughdadi, I. Adali, F. Manoudi

**Affiliations:** Psychiatry, Mohammed VI university hospital, Marrakech, Morocco

## Abstract

**Introduction:**

Substance use disorders (SUD) and behavioral addictions constitute a complex group of affections with a high prevalence around the world and in Morocco. Their treatment hindered by the frequency of relapses. Virtual reality (VR) is a computer-generated simulation, using specific hardware, that allows users to feel present in a virtual environment through various sensory stimuli. Thanks to its unique ecological validity, VR has gotten a lot of interest as a potential solution to craving assessment, but also as a tool that may be used in cue exposure therapy (CET) and/or cognitive behavioral therapy (CBT).

**Objectives:**

This study aims to present an overview of the use of VR in the field of addiction.

**Methods:**

The literature review was based on PubMed research using the key words: virtual reality, addictive, addiction, substance, alcohol, eating disorder, cocaine, cannabis, opioid, tobacco, nicotine, methamphetamine, gaming, gambling. Out of the 170 results initially found, 26 studies were included after screening.

**Results:**

Nicotine: significant signs of craving were induced by virtual smoking cues. Crushing virtual cigarettes was shown to reduce nicotine addiction, and virtual environment CET as a treatment method was better than classical pictures watching and as good as CBT.

Alcohol: VR systems were able to induce alcohol craving using both contextual and sensory alcohol-related cues. High levels of presence were reported thus proving ecological validity. There was a decrease in cue-elicited craving after virtual environment CET, with one study showing better results using VR CET than general cognitive therapy.

Methamphetamine: VR drug-related cues led to increased craving and skin conductance. VR based therapies are able to suppress cue-induced reactivity after multiple sessions.

Gaming: An increase in craving was observed when patients were exposed to game-related cues in a virtual environment. Similar results to CBT were reported with VR treatment.

Gambling: Casino-related cues were able to generate craving in gamblers. Multiple sessions based on repeated exposure and relaxation training led to a reduced urge to gamble.

Cannabis: Cannabis virtual environments caused a higher craving sensation than the neutral ones.

Cocaine: Significant increase in craving and heart rate during cocaine-related virtual scenes compared to baseline.

Eating disorders: Overall, VR CET showed better results than CBT in the treatment of bulimia nervosa and binge-eating disorder.

**Conclusions:**

The studies reviewed in this work showed promising results and were able to prove that VR could be a powerful tool in the field of SUD thanks to its several benefits in both assessment and treatment. Future work involving researchers in both VR and SUD domains and associating it to other technologies could expand the future prospects of the virtual world.

**Disclosure of Interest:**

None Declared

